# Risky business: rhesus monkeys exhibit persistent preferences for risky options

**DOI:** 10.3389/fpsyg.2014.00258

**Published:** 2014-04-24

**Authors:** Eric R. Xu, Jerald D. Kralik

**Affiliations:** Department of Psychological and Brain Sciences, Dartmouth CollegeHanover, NH, USA

**Keywords:** risk, decision-making, choice, reward value, gambling, primates

## Abstract

Rhesus monkeys have been shown to prefer risky over safe options in experiential decision-making tasks. These findings might be due, however, to specific contextual factors, such as small amounts of fluid reward and minimal costs for risk-taking. To better understand the factors affecting decision-making under risk in rhesus monkeys, we tested multiple factors designed to increase the stakes including larger reward amounts, distinct food items rather than fluid reward, a smaller number of trials per session, and risky options with greater variation that also included non-rewarded outcomes. We found a consistent preference for risky options, except when the expected value of the safe option was greater than the risky option. Thus, with equivalent mean utilities between the safe and risky options, rhesus monkeys appear to have a robust preference for the risky options in a broad range of circumstances, akin to the preferences found in human children and some adults in similar tasks. One account for this result is that monkeys make their choices based on the salience of the largest payoff, without integrating likelihood and value across trials. A related idea is that they fail to override an impulsive tendency to select the option with the potential to obtain the highest possible outcome. Our results rule out strict versions of both accounts and contribute to an understanding of the diversity of risky decision-making among primates.

## INTRODUCTION

Our lives are filled with choices, and many choice options involve an element of uncertainty or risk. In principle, people should prefer options with the highest expected value, but in practice, different choices often prevail. Expressed in economic terms, people do not consistently maximize utility. The reason appears to involve the difficulty in computing utility, especially in natural conditions, when options are many and the outcomes uncertain or risky. We focus here on risky options, and we broadly define an option’s risk level as the variation in its potential reward outcomes (Weber et al., 2004; McCoy and Platt, 2005; Hayden et al., 2008). Thus, an option with more than one potential outcome is risky and contains inherent opportunity costs based on the missed opportunities of the unchosen options.

Consider the calculations required to compare a safe option to a risky one. A safe option could involve receiving $1 for every choice. A risky option with the same average value could involve receiving $2, but only for half of the choices, with the other half yielding nothing. The expected utility of the risky option is a function of the potential outcome values and their likelihoods: ($2 × 0.5) + ($0 × 0.5) = $1. It is easy to see how such calculations could become computationally demanding with, for e.g., multiple choice options, each with multiple possible outcomes with different likelihoods.

Because of this computational complexity, our decision-making mechanisms have evolved to manage it using heuristics that substitute computationally difficult calculations with approximations (Tversky and Kahneman, 1974, 1981; Kahneman et al., 1982; Kahneman and Frederick, 2002; Chen et al., 2006; Kahneman, 2011; Lakshminarayanan et al., 2011; Kralik et al., 2012). To understand how we make decisions, therefore, it is not sufficient to evaluate precise outcomes; we must also understand the operations of the cognitive processes underlying these heuristics.

In general, people are risk averse and avoid options that may result in a perceived loss of a “lesser” gain, no gain at all, or an actual loss. In addition, when outcome values and likelihoods are difficult to integrate, people tend to focus on specific components, such as the potential perceived losses or missed opportunities (opportunity costs), while ignoring others, such as likelihoods. Because of such factors, how problems are posed affects decision-making (Tversky and Kahneman, 1974, 1981; Kahneman and Tversky, 1984; Kahneman, 2011). For example, people’s preferences are influenced by whether the outcome contingencies are presented to them in oral or written form, as opposed to those discerned through experience. Choices are also affected by the number of decisions to be made, such as one opportunity to gamble vs. multiple opportunities (Montgomery and Adelbratt, 1982; Klos, 2005; Sundali and Croson, 2006; Rabin and Vayanos, 2010; Kahneman, 2011). In one-shot gambles, people appear to be particularly averse to the potential perceived loss and thus tend to select the safer option. With repeated gambles, people take on more risk, believing that the potential for possible perceived loss is mitigated as actual outcomes converge to the expected utilities (which reflects another bias, known as the Samuelson fallacy; Samuelson, 1963; Montgomery and Adelbratt, 1982; Klos, 2005; Sundali and Croson, 2006; Rabin and Vayanos, 2010; Kahneman, 2011).

Here we focus on experiential problems (as opposed to oral or written ones), which characterizes many problems faced by humans and all of those faced by other animals. Such problems are less susceptible to certain biases, but can still be difficult to evaluate. We also take a comparative perspective, which can provide some insight into the evolutionary history of decision-making processes and can also clarify possible advantages conferred by different types of risk and value assessments under different environmental conditions, independent of language-related and sociocultural factors. We use “risk preference” or “risk-seeking” for a preference for risky over safe options, and “risk aversion” or “safety preference” for *vice versa*. 

There is evidence for a risk preference in rhesus monkeys (*Macaca mulatta*), a representative catarrhine monkey and a descendant of the last common ancestor of Old World monkeys, apes, and humans. For choices between options of equal overall expected utility, rhesus monkeys have been shown to have a preference for risky options (McCoy and Platt, 2005; Hayden and Platt, 2007; Hayden et al., 2008; O’Neill and Schultz, 2010). For example, they preferred a risky option with a 50/50 chance of either 250 or 50 microliters of fruit juice to a safe option that offered 150 microliters 100% of the time. Recently, however, the generality of this conclusion has been questioned. Heilbronner and Hayden (2013) suggested that the risk preference of rhesus monkeys might be context dependent, resulting from specific task parameters that might promote risk-seeking. These parameters include small differences in fluid reward, repeated gambles across hundreds, if not thousands, of trials, and short intertrial intervals (ITI) of a few seconds or less. In fact, a recent study found rhesus monkeys to be mildy risk averse (i.e., the percent of risky choices was less than 50%, i.e., risk neutrality, but not significantly so; Yamada et al., 2013). In this study, rhesus monkeys chose between a certain option that paid off a fixed amount of juice 100% of the time versus a risky option that paid off a particular amount 50% of the time, and nothing 50% of the time. Four conditions were used in which the amount of the safe option was changed. Within each condition five different risky options were used, each of different amounts, and thus the expected utility varied among these five risky options. In only the middle risky option in each condition were the expected utilities of the safe and risky options equal.

It remains unclear exactly why the risk preferences were different in this study versus the others that found a risky preference. Possible explanations include (1) higher amounts of juice used; (2) non-rewarded trials on 50% of risky choices; (3) significant experience with a fixed probability structure for the risky options and multiple different values that could have trained the monkeys to use both probability and value and thus expected utility; and (4) pie charts displayed at the beginning of the trial to represent value that may have helped simplify (e.g., linearize) the value estimation. 

To help delineate the key factors, we modified several of them while maintaining equal expected utilities in Experiments 1–6 as in the original studies. After first determining the relative value of three qualitatively different food items, we conducted seven short experiments using them as reward. Compared to the previous studies, we increased the stakes by using fewer trials per session, a longer intertrial interval, larger reward amounts, and greater variation in the risky outcomes. In addition, rather than fluid reward, our study used food items. The risky options also included non-rewarded outcomes (Yamada et al., 2013). In the first six experiments, the monkeys preferred the risky option. Finally, we tested whether their risk preference was due to a focus on the highest outcome payoff of the risky option (a salience bias), with neglect of likelihoods. Both monkeys exhibited sensitivity to the likelihoods, reversing their preferences to the safe option when it paid off four times more than the risky one.

## MATERIALS AND METHODS

### SUBJECTS

Two male rhesus monkeys were available for this study, denoted as Monkey T and P, ages 11 and 9, respectively. Animal care and use complied with all current laws, regulations, policies, and guidelines of the United States, the United States Department of Agriculture (USDA), the Public Health Service (PHS), and all procedures were approved by the Institutional Animal Care and Use Committee (IACUC) of Dartmouth College. The Center for Comparative Medicine and Research (CCMR) at Dartmouth maintains a full-time animal care and veterinary staff that monitors the monkeys’ daily health and well-being.

The monkeys were housed in a homeroom with automatically regulated temperature, ventilation, humidity, and lighting. The monkeys were intermittently housed together and individually: at times when they engaged in confrontations, which is normal periodic behavior in young rhesus macaque males of similar size and temperament (Thierry et al., 2004), the two monkeys were separated and individually housed for their safety. When pair-housed, they had direct physical contact with each other, and when individually housed, through a mesh grading divider between their cages. They also had direct visual contact with the other monkeys in the colony, as well as the animal care staff and experimenters. In addition, environmental enrichment included two or more enrichment items in their home cages at all times, daily playing of radio or videos in the room (the latter via a monitor mounted in view of all individuals), and regular access to a larger enrichment cage (172.7 cm × 96.5 cm × 182.9 cm) in an adjacent room. The monkeys had *ad libitum* access to food and water during the study.

### MATERIALS AND TESTING PROCEDURES

In the test room, the monkey’s chair was placed on the opposite side of a table, 76.2 cm, from the experimenter. A monkey sat in the chair, with his left arm loosely restrained (using two custom-made metal rings attached to the chair around both the upper and lower arms) and the right arm free to reach.

**FIGURE 1:**
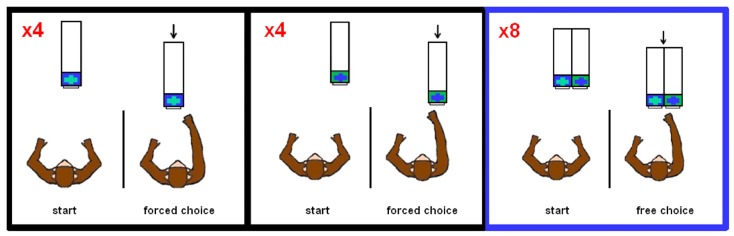
**Task design schematic of Experiments 1–7.** Each block consisted of four forced choice trials on each side (order randomly determined) followed by eight free choice trials. For each trial, the Plexiglas apparatus was presented to the monkey out of reaching distance before it was moved forward for selection.

In food preference testing, the monkeys were presented with two rectangular compartments (each 7.62 cm × 8.89 cm), which had transparent lids in order to see the food items directly. In Experiments 1–7, which tested risk preferences, the monkeys were presented with one or two opaque compartments, also 7.62 cm × 8.89 cm, which might or might not contain food items. For these seven experiments, the left compartment lid was blue with a green center cross, and the right compartment lid was green with a blue center cross (see **Figure 1**).

Each compartment was glued to its own underlying Plexiglas® sheet (12.7 cm × 35.56 cm) with the compartment at the monkey’s end of the sheet. The compartments could thus be moved both simultaneously (with the sheets touching on adjacent edges) and independently. An opaque plastic divider separated the experimenter and the monkey. An opening at the bottom of the divider allowed the experimenter to present both compartments simultaneously by placing each hand on the back of a Plexiglas sheet and sliding the compartments toward the monkey. The monkeys made selections by lifting a compartment lid to obtain the contents inside.

To minimize the potential for inadvertent cues from the experimenter, several procedures were implemented: (a) as just mentioned, an opaque plastic divider separated the experimenter and the monkey, thus preventing the monkey from seeing the face and upper body of the experimenter; (b) white noise was played to minimize auditory distractions; and (c) the experimenter followed a well-practiced routine on each trial.

For all testing in the study (food preference tests and experiments), each trial began with the experimenter holding the Plexiglas apparatus so that both compartments were 40.6 cm in front of the monkey but not within the monkey’s reach. The experimenter held this position until the monkey looked at the compartments, as determined by monitoring the monkey via a close-circuit camera and video monitor. Next, both compartments were pushed forward to a fixed position within the monkey’s reach. Once the monkey touched the lid of one of the compartments, the unselected compartment was quickly removed. The intertrial interval throughout the study was approximately 15 s.

To rule out potential side biasing effects in food preference testing, the food item positions (left or right compartment) were randomized within a daily session, with the constraint that each food item was presented to the left and right an equal number of times in the session. For each monkey, 60 trials were conducted per daily session, and two sessions were conducted for each of the three food preference testing conditions.

For risk preference testing (Experiments 1–7), we conducted the trials in blocks, with each block consisting of 16 trials: eight forced choice trials – four forced choice trials from one of the two compartments (randomly determined) followed by four forced choices from the other compartment – and eight free choice trials (**Figure 1**). The monkeys typically performed four blocks per daily session with 12 blocks per experiment (further detail below). The experiments were conducted in consecutive order and only one experiment was conducted in any daily session (thus, on sessions in which an experiment was completed, the session terminated, and the new experiment was begun the following session). The experiment the monkey experienced in each daily session was not cued; rather, we used the forced choice trials conducted at the beginning of every block as the means by which the monkeys learned the contingencies of the given experiment. **Table 1** summarizes the outcome contingencies for both the risky and safe options for each experiment, along with (a) the coefficient of variation (CV) as a measure of the level of risk, i.e., the standard deviation divided by the mean, and (b) the objective utility, i.e., the sum of the products of value by likelihood for all option outcomes, of the risky options.

**Table 1:** The reward contingencies in the seven experiments (A: mini M&M, B: mini marshmallow, and C: cheerio).

Experiment	Safe option	Risky option		
		Contingencies	CV	Utility
1	1B (100%)	2B (50%) and 0 (50%)	1	1B
2	1A (100%)	2A (50%) and 0 (50%)	1	1A
3	1A (100%)	4A (25%) and 0 (75%)	2.2	1A
4	1A (100%)	2A (50%) and 0 (50%)	1	1A
5	1A (100%)	8A (12.5%) and 0 (87.5%)	5	1A
6	1C (100%)	2C (50%) and 0 (50%)	1	1C
7	1A (100%)	2A (12.5%) and 0 (87.5%)	5	0.25A

*CV: coefficient of variation of the risky option; Utility: the expected mean value of the risky option by objective calculations.*

To minimize the potential for the actual experienced payoffs associated with the risky option deviating significantly from the assigned contingencies, the outcomes of the risky options were set up in the following way. We first describe the free choice trials. In all experiments, the outcomes associated with the risky option occurred according to the assigned proportions across each block of eight consecutive free choice trials. Thus, for example, in Experiment 1 four of the eight free choice trials were pseudo-randomly assigned to pay off two mini marshmallows; and thus every free choice block of eight trials paid off exactly as listed in **Table 1**. We next describe the forced choice trials. For all experiments except for 5 and 7, the outcomes associated with the risky option occurred according to the assigned proportions across each block of four consecutive forced choice trials. Thus, for example, in Experiment 1 two of the four forced choice trials were pseudo-randomly assigned to pay off two mini marshmallows. For Experiments 5 and 7, the outcomes associated with the risky option occurred according to the assigned proportions across every two blocks, and thus across every eight forced choice trials. Thus, for example, in Experiment 5 one of the eight forced choice trials across every two blocks (i.e., four in the first block, four in the second) was pseudo-randomly assigned to pay off eight mini M&Ms. As reported in the section ***Potential response strategies,*** we found no evidence that the monkeys were sensitive to the local changes in the trial-by-trial outcome probabilities due to this baiting procedure.

To rule out the potential influence of location biases, each experiment was conducted in two configurations in which the safe and risky option switched compartment positions. The compartments retained their same color and shape features, and thus the monkeys had to discern the flipped contingencies based on the forced choice trials. In Experiment 1, Monkey P was run on 11 blocks in one configuration and 10 blocks in the other, while Monkey T was run on eight blocks in each configuration. In Experiment 2, Monkey P was run on eight blocks in one configuration and six blocks in the other, while Monkey T was run on 12 blocks in one configuration and 11 blocks in the other. In Experiments 3–7, both monkeys were run on 12 blocks in each configuration. To obtain the monkeys’ stable choice preference in each experiment, we analyzed the last 30 free choice trials in both configurations combined, i.e., 60 total trials per experiment. All binomial tests were two-tailed.

## RESULTS

In a series of experiments in which the mean utilities between the safe and risky options were equivalent, we manipulated the value of the gamble via changes in reward quality (Experiments 1, 2, 4, 6), the risk level via changes in the CV (Experiments 2–5), and novelty (Experiment 4 replicating Experiment 2). Despite these changes, both monkeys continued to prefer the risky over the safe option. Only when the utility associated with the safe option was greater than that for the risky option did the monkeys prefer the safe option (Experiment 7).

### FOOD PREFERENCE

We first conducted food preferences to assess the relative value of food items that differed in quality. We tested three food items using a two alternative free choice procedure: a mini M&M® (food A), a mini marshmallow (food B), and a cheerio® (food C). The monkeys were familiar with these food items, but only received them during the study in the food preference tests and experiments.

#### Food preference results

For mini M&Ms (A) vs. cheerios (C), both monkeys exhibited a significant preference for the former (Monkey P, 99.2%, binomial test, *z* = 10.68, *p* < 0.001; Monkey T, 90.8%, binomial test, *z* = 8.85, *p* < 0.001). They also exhibited a significant preference for mini M&Ms (A) over mini marshmallows (B) (Monkey P, 64.2%, binomial test, *z* = 3.01, *p* < 0.01; Monkey T, 73.3%, binomial test, *z* = 5.02,* p* < 0.001) and for mini marshmallows (B) over cheerios (C) (Monkey P, 90.8%, binomial test, *z* = 8.85,* p* < 0.01; Monkey T, 80.0%, binomial test, *z* = 6.48,* p* < 0.001).

#### Food preference discussion

Overall, both monkeys exhibited a clear transitive preference among the three food items (A > B > C). They valued mini M&Ms the most, mini marshmallows second, and cheerios least. Neither the food’s mass, caloric value, nor sugar content fully accounted for these preferences. One mini M&M had a mass of 0.30 g (0.19 g sugar) and 1.47 cal; one mini marshmallow had 0.50 g (0.28 g sugar) and 1.67 cal; and one cheerio had 0.10 g (0.03 g sugar) and 0.39 cal. So the preferred subjective values did not fall in the same order as the objective values, although cheerios came out last in both. In terms of size (volume), the preferred food, mini M&Ms, was the smallest of the three foods. Thus, although mini marshmallows were the largest, heaviest, most caloric, and contained the most sugar, the monkeys preferred the mini M&Ms, perhaps because of its chocolate content or its higher sugar density [mini M&Ms (A): 1.06g/mm^3^; mini marshmallows (B): 0.56g/mm^3^; cheerios (C): 0.33g/mm^3^]. Finally, the food preference results also show that even though the monkeys were not food restricted, they were sufficiently motivated to select and consume the items, and they held clear preferences among the items.

### EXPERIMENT 1

All trials consisted of a safe option in one compartment and a risky option in the other. The safe option always contained one mini marshmallow, while the risky option yielded two mini marshmallows 50% of the time and nothing 50% of the time. The mean utility of both options was 1 mini marshmallow.

#### Experiment 1 results

**FIGURE 2:**
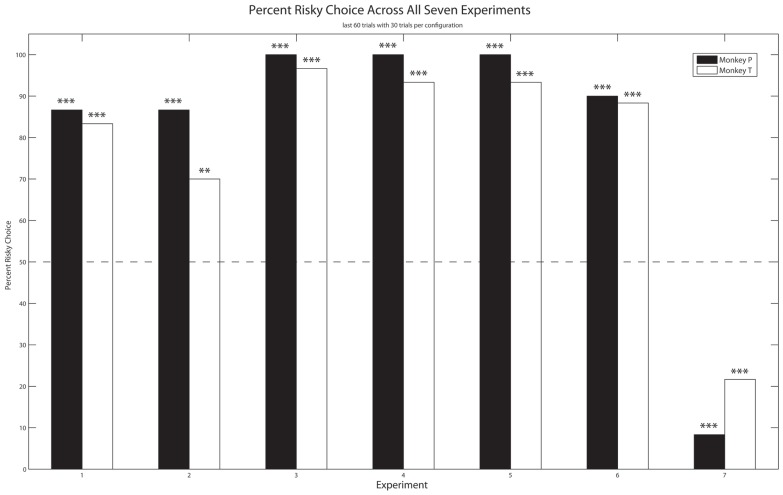
**Experiment 1–7: The mean percentage of trials each monkey selected the risky option over the last 30 free choice trials in both configurations (60 total trials).** Dashed line: no preference; Asterisks: significant preference (two-tailed binomial test, **p < 0.01, ***p < 0.001).

Both monkeys exhibited a significant preference for the risky option over the safe option (**Figure 2**). Monkey P selected the risky option at an 86.7% rate (binomial test, *z* = 5.55, *p* < 0.001) and Monkey T did so at an 83.3% rate (binomial test, *z* = 5.03,* p* < 0.001).

#### Experiment 1 discussion

Both monkeys preferred the risky option over the safe one when foraging for mini marshmallows, the intermediately valued food item. McCoy and Platt (2005) found a risk preference using time access to fluid rewards as their measure, and our results generalize this finding to discrete food quantities. Experiment 1 also differs from previous ones in that our monkeys exhibited a risk preference with relatively few trials and a longer ITI (McCoy and Platt, 2005; Hayden and Platt, 2007; Hayden et al., 2008; O’Neill and Schultz, 2010), thus further generalizing the finding.

We can only infer the subjective value of food items, although the monkeys appear to place a high value on the marshmallows. We cannot rule out the possibility that their motivation was insufficient to be affected by the “0” trials of the risky option, i.e., the trials on which the monkeys obtained no marshmallows. In Experiment 2, we used mini M&Ms, a more valued food item, to determine if increasing the intrinsic reward value would change the monkeys’ risk preference.

### EXPERIMENT 2

*Subjects, Materials,* and* Methodology* were the same as in Experiment 1 except for the food used: mini M&Ms.

#### Experiment 2 results

As **Figure 2** shows, both monkeys exhibited a significant preference for the risky over the safe option. Monkey P and T selected the risky option 86.7% (binomial test, *z* = 5.55, *p* < 0.001) and 70.0% (binomial test, *z* = 2.97,* p* < 0.01) of the time, respectively.

#### Experiment 2 discussion

The more valuable food item, mini M&M (A), did not alter the monkeys’ general risk preference as compared to mini marshmallow (B), the intermediately valued food item used in Experiment 1. Thus, the intrinsic reward value difference between these two food items did not have a significant effect on the monkeys’ general risk preference. We test reward quality again in Experiment 6.

### EXPERIMENT 3

In McCoy and Platt’s (2005) study, the monkeys’ risk preference increased with CV when it was progressively increased from 0.0667 to 0.667. In our Experiments 1 and 2, the CV for the risky option was 1. It is unclear whether risky behavior would continue when the risky option’s CV becomes >1. In addition, the likelihoods of the outcomes in the risky options used in previous monkey studies (McCoy and Platt, 2005; Hayden and Platt, 2007; Hayden et al., 2008; O’Neill and Schultz, 2010) have been 50–50%. In such cases, the high outcome has a relatively high possibility per trial. In Experiment 3, we addressed these issues by increasing the risky option’s CV above 1 and lowering the likelihood of the high reward outcome.

*Subjects, Materials,* and* General Methodology* were the same as in Experiment 1. All trials consisted of a safe option in one compartment and a risky option in the other. The safe option always contained one mini M&M, while the risky option yielded four mini M&Ms 25% of the time and nothing the remainder of the time (**Table 1**). Thus, the CV of the risky option was 2.2, and the mean utility of both options was 1 mini M&M.

#### Experiment 3 results

Both monkeys continued to exhibit a significant preference for the risky option over the safe option, and did so to a greater extent than in Experiments 1 and 2 (**Figure 2**). Specifically, Monkey P and T selected the risky option 100% (binomial test, *z* = 7.62,* p* < 0.001) and 96.7% (binomial test, *z* = 7.1,* p* < 0.01) of the time, respectively.

#### Experiment 3 discussion

Both monkeys strongly preferred the risky option over the safe option with the new experimental contingencies. Hence, the risk preference persisted despite increasing the CV of the risky option from 1 to 2.2, reducing the likelihood of the highest outcome from 50% to 25%, and increasing the highest outcome from two discrete mini M&Ms to four. The results suggest that the monkeys might be more attentive to the large reward outcome and/or the outcome’s CV, although further testing is needed to assess the generality of this conclusion.

### EXPERIMENT 4

The risk preference observed in Experiment 3 might have been due to the novelty of the risky option’s new reward contingency compared to Experiment 2 (with the safe option remaining the same, but the risky option contingency changing). We do not believe this to be the case for two reasons: (1) our analysis used the last 30 trials in each configuration after sufficient learning and a stable preference was obtained; and (2) in Experiments 1 and 2 both the safe and risky option contingencies were novel, and yet the monkeys exhibited preferences for the risky option. However, we nonetheless addressed this issue in Experiment 4 by repeating Experiment 2 to test whether the risky option’s novelty in Experiment 3 compared to Experiment 2 led to the risky choice preference in Experiment 3. Furthermore, to standardize the block sizes across configurations and subsequent experiments, we set the block size at 12 per configuration beginning in Experiment 3. We therefore also repeated Experiment 2 to test the contingencies under the standardized protocol to enable direct comparisons across experiments (see ***Effects of contingency parameters***).

The methods for Experiment 4 were the same as described in Experiment 2.

#### Experiment 4 results

Both monkeys exhibited a significant preference for the risky option, as they had in Experiment 2 (**Figure 2**), at a rate of 100% for Monkey P (binomial test, *z* = 7.62, *p* < 0.001) and 93.3% for Monkey T (binomial test, *z* = 6.58, *p* < 0.001).

#### Experiment 4 discussion

Both monkeys continued to prefer the risky over the safe option. Thus, the risk preference of the monkeys observed in Experiment 3 appeared to be a general preference rather than one based on the novelty of the risky option’s contingency in Experiment 3 compared to Experiment 2. In addition, we replicated the general risky preference exhibited in Experiment 2.

### EXPERIMENT 5

Because the monkeys’ risky option preferences did not seem to be affected by the novelty of the risky option’s contingencies, we explored more extreme parameters.

In Experiment 5, the safe option always contained one mini M&M, while the risky option yielded eight mini M&Ms 12.5% of the time and nothing the remainder of the time. Thus, the CV of the risky option was 5, and the mean expected value of both options was 1 mini M&M.

#### Experiment 5 results

As in the previous experiments, both monkeys exhibited a significant preference for the risky option over the safe option (**Figure 2**), at a rate of 100% (binomial test, *z* = 7.62,* p* < 0.001) for Monkey P and 93.3% (binomial test, *z* = 6.58,* p* < 0.001) for Monkey T, much like Experiments 3 and 4.

#### Experiment 5 discussion

Despite the three changes from Experiment 3 – increasing the CV of the risky option from 2.2 to 5, reducing the likelihood of the risky option’s positive outcome from 25 to 12.5%; and increasing the risky option’s positive reward outcome from four discrete mini M&Ms to eight – the monkeys reliably preferred the risky option. This result also serves as a control for a CV of 5, which applies to Experiment 7 as well. We take up this point later.

### EXPERIMENT 6

An important issue is the extent decision-making preferences change based on one’s intrinsic motivation level. In principle, motivation relates to both the current state of the organism, i.e., their level of “wealth,” and the current state of the environment, whether rich or lean. Results thus far are mixed, with individuals appearing to become more and less risk-seeking with increasing motivation in different studies (Caraco et al., 1980; Kacelnik and Bateson, 1996, 1997; Abreu and Kacelnik, 1999; Yamada et al., 2013). In Experiment 6 we tested whether a decrease in the motivation level might result in a preference reversal to the safe option by decreasing the intrinsic value of the food reward. To do so, we repeated the outcome contingencies of Experiments 1, 2, and 4 but replaced the food items with cheerios, the least preferred food.

The safe option always contained one cheerio, while the risky option yielded two cheerios 50% of the time and nothing 50% of the time (**Table 1**).

#### Experiment 6 results

Monkey P and T selected the risky option 90% (binomial test, *z* = 6.07,* p* < 0.001) and 88.3% (binomial test, *z* = 5.81,* p* < 0.001) of the time, respectively (**Figure 2**).

#### Experiment 6 discussion

Both monkeys preferred the risky over the safe option using cheerios as reward. Thus, a change in reward quality did not change the overall risk preferences of the monkeys, at least within the range tested in our study. However, evidence was found for some effect of intrinsic reward value (see ***Effects of contingency parameters***).

### EXPERIMENT 7

A possible account for the risk preference in Experiments 1–6 was that the monkeys might be driven toward the option with the highest possible reward outcome (a salience bias). Along with this possibility, the monkeys might also be relatively insensitive to the likelihood-based valuations of the options. This could be due to the stakes in which there is no objective loss other than opportunity costs.

To test these possibilities, in Experiment 7 the monkeys chose between a safe and a risky option with the risky option having a lower mean utility compared to the safe one. The risky option still had potential for the largest reward outcome in the experiment, as in Experiments 1–6.

The safe option always contained one mini M&M, while the risky option yielded two mini M&Ms 12.5% of the time and nothing the remainder of the time. Thus, the CV of the risky option was 5 (as in Experiment 5) and its mean expected utility was 0.25 mini M&Ms. As in Experiments 2–5, the mean expected utility of the safe option was 1 mini M&M, four times larger than the risky option in this experiment (**Table 1**).

#### Experiment 7 results

Unlike the results of Experiments 1–6, both monkeys exhibited a significant preference for the safe option over the risky one (**Figure 2**). Specifically, Monkey P selected the risky option at a rate of 8.3% (binomial test, *z* = -6.33,* p* < 0.001) and Monkey T did so at 21.7% (binomial test, *z* = -4.26,* p* < 0.001).

#### Experiment 7 discussion

These results show that neither monkey simply chose the option that yielded the most valuable possible outcome, which would have reflected a salience bias. The results further show that both monkeys integrated likelihood and the reward value across trials to make their decisions.

### COMPARISONS ACROSS EXPERIMENTS

#### Effects of contingency parameters

Because Experiments 1 and 2 were conducted with fewer than 12 blocks per condition, we compared the percent of risky choices in Experiments 3–7. A comparison of Experiments 4 and 6 (**Table 1**) showed that there was an effect of reward quality with the percent risky choice decreasing with a decrease in reward quality in both monkeys; however, the decrease was significant only for Monkey P [*X*^2^(1) = 6.316,* p* = 0.012; Monkey T, *X*^2^(1) = 0.901,* p* = 0.343]. Thus, to the extent overall reward quality influences motivation, there was some evidence for greater motivation leading to increased risk-preferring behavior. A comparison of Experiments 3 and 4 [Monkey P, *X*^2^(1) = 0,* p* = 1; Monkey T, *X*^2^(1) = 0,* p* = 1], Experiments 4 and 5 [Monkey P, *X*^2^(1) = 0,* p* = 1; Monkey T, *X*^2^(1) = 0,* p* = 1], and Experiments 3 and 5 [Monkey P, *t*(2) = 3.28, *p* = 0.08; Monkey T, *t*(2) = 0.35, *p* = 0.76] found no relationship between CV and the percent of risky choices for either monkey, which could have been due to a ceiling effect. Finally, a comparison of Experiments 4 and 7 showed that likelihood affected the percent of risky choices in both monkeys [Monkey P, *X*^2^(1) = 127.03,* p* < 0.001; Monkey T, *X*^2^(1) = 60.92,* p* < 0.001], and thus preferences could not be explained by a neglect of the non-rewarded outcomes of the risky options.

#### Potential response strategies

Although there was some evidence for persistence or response habits when the configurations changed, nonetheless, in every experiment, both monkeys changed their choice to the risky (Experiments 1–6) or safe option (Experiment 7). Even so, strategies such as “win-stay, lose-shift” or “win-shift, lose-stay” were possible (Genovesio et al., 2005; Heilbronner and Hayden, 2013). Due to our baited procedure for determining the outcomes of the risky options (see **MATERIALS AND TESTING PROCEDURES**), a “win-shift, lose-stay” strategy would be a sign that the monkeys were tracking the changes in the trial-by-trial probabilities instead of showing a true overall risk preference. However, we found no evidence for this response strategy by either monkey in any experiment. Furthermore, we found no evidence for the “win-stay, lose-shift” response strategy in any experiment except for one monkey in Experiment 7, when mean utilities between options were unequal. In all other cases, comparing the proportion of trials each monkey stayed with the risky option after receiving the largest outcome with the proportion each monkey stayed with the risky option when he did not receive the largest outcome, we found no significant differences [Experiment 1: Monkey P, *X*^2^(1) = 0.15, *p* = 0.70; Monkey T, *X*^2^(1) = 0.08, *p* = 0.77; Experiment 2: Monkey P, *X*^2^(1) = 0.02, *p* = 0.89; Monkey T, *X*^2^(1) = 3.04, *p* = 0.08; Experiment 3: Monkey P, *X*^2^(1) = 0.09, *p* = 0.77; Monkey T, *X*^2^(1) = 0.75, *p* = 0.39; Experiment 4: Monkey P, *X*^2^(1) = 1.94, *p* = 0.16; Monkey T, *X*^2^(1) = 1.10, *p* = 0.29; Experiment 5: Monkey P, *X*^2^(1) = 0.54, *p* = 0.46; Monkey T, *X*^2^(1) = 3.04, *p* = 0.08; Experiment 6: Monkey P, *X*^2^(1) = 0.41, *p* = 0.52; Monkey T, *X*^2^(1) = 0.01, *p* = 0.91; Experiment 7: Monkey P, *X*^2^(1) = 1.24, *p* = 0.276]. The exception was Monkey T in Experiment 7, in which he appeared to exhibit a “win-stay, lose-shift” strategy with the risky option (*X*^2^ = 4.22, *p* = 0.04). Thus, except for Monkey T in Experiment 7, the response strategy that best reflected their choice behavior was “win-stay, lose-stay,” suggesting that the monkeys were exhibiting an overall risk preference for Experiments 1–6.

In Experiments 5 and 7, in the forced choice trials, the outcomes associated with the risky option occurred according to the assigned proportions across every two blocks, and thus across every eight forced choice trials (i.e., four forced choice trials in one block, and four in the second). This baiting procedure resulted in trial blocks in which one of the risky forced choices was rewarded, followed by a trial block in which none of the risky forced choices were rewarded (or *vice versa*). If the monkeys were sensitive to the local changes in the trial-by-trial outcome probabilities due to this baiting procedure, there should be a difference in the percent of risky choices in the blocks in which the large payoff was received in the forced choice trials vs. when no reward was received in the forced choice trials. To test for a potential pattern in Experiments 5 and 7, we examined the free choices in the last four blocks in each left–right configuration (eight blocks total). We compared the four blocks in which one of the four risky forced choices was rewarded to the four blocks in which none of the risky forced choices were rewarded. Neither monkey showed a significant behavioral difference between these blocks in either experiment [Experiment 5: Monkey P, *X*^2^(1) = 0, *p* = 1; Monkey T, *X*^2^(1) = 0.35, *p* = 0.55; Experiment 7: Monkey P, *X*^2^(1) = 0.22, *p* = 0.64; Monkey T, *X*^2^(1) = 1.46, *p* = 0.23], and thus they appeared to be following the general risky option contingency rather than local trial-by-trial changes.

#### Dynamics across experiments

**Table 2** shows the number of trials in which each monkey first exhibited his final preference in each left–right configuration (examined with a sliding window of 16 free choice trials). For example, in Experiment 1, Monkey P exhibited a significant risk preference after 75 free choice trials in his first experienced configuration, and then showed a significant risk preference after 66 free choice trials when the safe and risky options changed positions. We found no significant decrease (or increase) across all experiments either overall or for either configuration for either monkey (linear regression using the values in each column of **Table 2**, *p* > 0.05), suggesting that the monkeys were learning each new contingency for each experiment.

**Table 2:** The number of trials in which each monkey first showed his final preference, with preference measured using a sliding window of 16 consecutive free choice trials, and a preference-significance threshold of ≥13/16, (*p* < 0.05, two-tailed binomial test), assessed for each configuration separately.

Experiment	Monkey P			Monkey T		
	Conf. 1	Conf. 2	Total	Conf. 1	Conf. 2	Total
1	75	66	141	63	22	85
2	48	27	75	64	84	148
3	42	43	85	20	71	91
4	44	25	69	56	67	123
5	21	31	52	33	40	73
6	28	37	65	77	35	112
7	69	78	147	78	31	109

*Conf: configuration.*

Examining only the experiments with risk preferences, i.e., Experiments 1–6, neither monkey exhibited a significant change overall (linear regression, Monkey P: *R*^2^ = 0.63, *p* = 0.059; Monkey T: *R*^2^ = 0.13, *p* = 0.833), although Monkey P was trending toward significance (*p* = 0.059), which was due to a significant decrease for Configuration 1 (*R*^2^ = 0.8, *p* < 0.05) but not Configuration 2 (*R*^2^ = 0.28, *p* = 0.28). In addition, given identical reward contingencies in Experiments 2 and 4, a comparison of the acquisition rates to acquire a significant preference in the two experiments enabled a direct test of experience on choice behavior. The acquisition rates did not differ [Monkey P, *t*(2) = 0.21, *p* = 0.85; Monkey T, *t*(2) = 1.10, *p* = 0.39], providing evidence against an experiential effect across experiments. Finally, for both monkeys, the total number of trials to achieve significance in both configurations was the lowest (and thus the fastest acquisition rate) for Experiment 5, in which they could obtain the largest amount of the preferred food item on any risky trial – eight mini M&Ms – 12.5% of the time.

## DISCUSSION

### RISK PREFERENCE: EXPERIMENTS 1–6

The preferences for risky options reported thus far for macaque monkeys are potentially context driven, resulting from specific task parameters that might promote risk-seeking (Heilbronner and Hayden, 2013). Previous studies that found a risk preference in rhesus monkeys have used small reward amounts and differences, repeated gambles across hundreds of trials, and short intertrial intervals, all of which may promote risk-seeking behavior (Hayden and Platt, 2007, 2009; O’Neill and Schultz, 2010; Heilbronner and Hayden, 2013). In contrast, a recent study found mild risk aversion in rhesus monkeys (Yamada et al., 2013). Compared to the previous studies, this one had higher reward amounts, non-rewarded trials, multiple comparisons with unequal expected utilities, and visual cues (pie charts) to represent the available reward amounts. It thus remains unclear what factors lead to risky versus safe preferences in rhesus monkeys. In Experiments 1–6, we examined the effect of several factors while keeping the expected utilities of the choice options equal (as they were in the original studies that found preferences for the risky options). More specifically, we examined the following: (1) to change reinforcer type as well as to examine the effects of reward quality, we used food rather than fluid reward; (2) to increase the stakes with respect to the variation in the risky options, we heightened the risk level beyond those used in previous studies; (3) to increase the cost of risky choices, we (a) used no reward outcomes (omission of reward), and (b) reduced the likelihood of receiving the large reward outcome associated with the risky option to below 50%; and (4) to increase the significance of individual choices, we used (a) a maximum of 32 daily free choice trials per monkey (as opposed to hundreds of free choice trials), and (b) a longer intertrial interval (15 s). Despite these modifications, both monkeys showed a consistent preference for the risky option except when the mean utility for the safe option was greater than for the risky option, and in our case, fourfold greater. Future testing can systematically modify the mean utility between the safe and risky options to determine the indifference (subjective equivalence) point of such preferences.

Because fluid rewards were previously used (McCoy and Platt, 2005; Hayden and Platt, 2007; Hayden et al., 2008; O’Neill and Schultz, 2010), our results show that the monkeys’ risk preference extends to food-based rewards as well. In addition, Hayden and Platt (2007) found that the rhesus monkeys’ preference reached neutrality as the intertrial interval increased to 90 s in their gambling task. In our study, we used an intertrial interval of 15 s. However, because our risky option used reward omission outcomes instead of small-reward outcomes, the monkeys experienced a further delay similar to an additional intertrial interval. Because the large reward outcome had a lower likelihood (e.g., 12.5% in Experiment 5 compared to 50% in the study of Hayden and Platt), the monkeys easily experienced many interreward intervals >90 s; and yet they still maintained a risky preference.

One possible account for the risk preference of the monkeys in our study was that the initial preference for the risky option in Experiment 1 drew their preference toward the risky option for the subsequent Experiments 2–6. It has been shown that choices themselves can influence subsequent preferences (Montgomery and Adelbratt, 1982; Klos, 2005; Sundali and Croson, 2006; Rabin and Vayanos, 2010; Kahneman, 2011), especially when subjects must learn the outcome likelihoods (i.e., probabilities) via experience over trials. Although interactions among experiments could influence choices, we think that this factor was minimal in our study. First, we reversed the left–right configurations within each experiment while holding the visual stimulus locations constant to mitigate against simple stimulus–response associations driving choice. Thus the monkeys’ preferences needed to be based on the actual reinforcement contingency in each experiment independent of visual stimuli and location. Second, to help demarcate the change in the reinforcement contingencies across the experiments (and configurations within each experiment), we used a forced and free choice block structure in which the monkeys received four consecutive forced choice trials with each option, followed by eight free choice trials. The forced choice trials help provide clearer feedback with respect to the current reinforcement contingencies. Third, if the monkeys were continuing to use the same general likelihoods across experiments, the monkeys should not have required so many trials to develop a significant preference in Experiments 2–6: an average of 35 (Monkey P) and 55 (Monkey T) per configuration, 69 (Monkey P) and 109 (Monkey T) per experiment (**Table 2**); and further, they should have exhibited a clear decrease in the number of trials to attain a significant preference across Experiments 1–6, which they did not. Fourth, Experiment 4, which replicated Experiment 2, provided a test for the effect of experience. The acquisition rates did not differ for either monkey. Finally, the overall faster acquisition rate of both monkeys in Experiment 5 (which had the largest payoff outcome of the more-preferred food item), as well as the reversal of preference to the safe option in Experiment 7, showed that the monkeys were influenced by the actual reward contingencies of each experiment. Thus, the continued risk preferences appeared to reflect a preference for the given option under each of the experimental contingencies.

Thus, the risk preference generalizes across multiple contexts. Yet it remains unclear why a strong risk preference was found here, whereas a mildy risk averse preference was found in the Yamada et al. (2013) study. There appear to be three leading possibilities. One, because we baited our risky trials (see **MATERIALS AND TESTING PROCEDURES**), ensuring that the reward would occur within a certain number of trials, it is possible that the decreased variance affected decision-making by reducing the negative impact of the non-rewarded trials (although no direct impact on behavioral strategies was observed). Second, experience with fixed probabilities and multiple risky values might help to train the monkeys to respond to both probability and value and thus according to expected utility. Finally, the use of pie charts to represent the available reward amounts during decision-making might help to clarify and simplify the reward estimation. It certainly should help to minimize memory effects. Further work will be necessary to determine the contributing factors. 

Thus, taken together, contextual factors clearly influence decision-making preferences (Heilbronner and Hayden, 2013). Nonetheless, inherent preferences might still exist, leading to species and individual differences. Such potential biases need to be examined under minimal experience and with multiple measures including acquisition rates. It also will be critical to compare groups in testing paradigms as identical as possible. To that end, people were tested in a comparable paradigm to that used with rhesus monkeys that obtained risk-seeking in the monkeys (Hayden and Platt, 2009). The human subjects appeared to be less loss averse than in instructed tasks, with the majority of people being risk-neutral (i.e., no significant preference found between safe and risky options). In fact, three general populations could be identified: risk-averse, risk-neutral and risk-seeking. In addition, in a similar paradigm, differences were found between human children, adolescents, and young adults, with children showing a preference for risky over safe options, adolescents showing a mild risk preference, and young adults generally showing a preference for the safe option (Paulsen et al., 2011b, 2012). Thus, human risk preferences cannot be simply explained by a general context difference such as instruction- vs. experience-based choices.

It is of interest that rhesus monkey risk preferences may resemble those of some human children, adolescents, and adults, at least to some degree (Hayden and Platt, 2009; Paulsen et al., 2011a, b, 2012). It is worth exploring whether this finding results from similar relative differences in the balance of control of brain structures involved in higher-level integration of value across trials vs. those driving impulsive responses (McClure et al., 2004; Delgado et al., 2008; Hare et al., 2009; Hassin et al., 2010; Kober et al., 2010; Volkow, 2010; Volkow et al., 2010; Berkman et al., 2011; Goldstein and Volkow, 2011; Heatherton and Wagner, 2011). A preference for risky options in some non-human animals, human children, adolescents, and some human adults could reflect relatively weak executive control. 

More specifically, general risk preferences such as what we observed in Experiments 1–6 could be due to value estimation that is non-linear. That is, the actual value added per reward item might increase with an increasing number of items. In other words, two M&Ms might have more than twice the value of one M&M. Some evidence suggests that rhesus monkeys are significantly more sensitive to changes in high-reward outcomes compared to low-reward outcomes (Hayden et al., 2008). Our results agree. The fastest acquisition rate occurred in Experiment 5, in which the monkeys could obtain the largest amount of the preferred food item in any experiment – eight mini M&Ms – even though it was only received 12.5% of the time. Similarly, risk preferences could reflect an insensitivity to the lower-valued risky outcome, especially when the value of the outcome is lower than the potential “jackpot,” but nonetheless positive or 0, as opposed to a negative value associated with an aversive outcome. Risk preferences could also reflect a relative insensitivity to the likelihoods, and in consequence, to the risk being taken (Kahneman and Tversky, 1979; Kahneman, 2011). Finally, risky choice behavior could also reflect a preference for outcome stochasticity (Hayden et al., 2008). Future work will be needed to tease apart the potential influences on risky behavior such as a fixation on the highest outcomes, a relative insensitivity to non-rewarded or low cost outcomes, a relative neglect of likelihood, a preference for outcome stochasticity, and other factors.

As opposed to risk preferences reflecting poor behavioral control, another possibility is that a preference for risk evolved as an adaptation for exploratory behavior in foraging or social behavior. Risk preference and impulsiveness in rhesus monkeys could reflect ancestral conditions that selected for such behavior, such as an omnivorous diet that might provide a selective advantage for the seeking of new outcomes of foraging choices or the heightened conspecific competition in a rigidly hierarchical social system (Thierry et al., 2004; Evans and Beran, 2007; Wise, 2008; Kralik et al., 2012; Passingham and Wise, 2012).

At the same time, there is certainly diversity and individual variability in non-human primates and people, and selection forces sculpting decision-making strategies may have led to multiple response “niches,” with relatively bolder and more prudent individuals both finding success. Indeed, even our two monkeys exhibited differences in their choice behavior: e.g., the effect of reward quality, and the use of response strategies in Experiment 7. Nonetheless, general species differences are also possible. Because our two subjects revealed the same general preferences in all seven experiments and because our results match other studies showing risk preferences in rhesus monkeys (McCoy and Platt, 2005; Hayden et al., 2008), our findings might represent rhesus monkey preferences in general, at least under our task parameters. Future work with more subjects will be necessary to determine species-level risky preferences and to further characterize the circumstances they may manifest. A two-subject study does however reveal a capacity within the species among at least a subpopulation of individuals, especially when corroborated by other published findings.

Finally, we found some evidence for the influence of reward quality on preference: risk preferences decreased with the decrease in reward quality in Experiment 6 versus 4 (significantly so for monkey P). A heightened general reward level in the environment can increase the motivational level of the individual, leading to increased response vigor (Niv et al., 2007). This finding corroborates those that have found increasing risk-seeking with increasing motivation, such as hunger, but contrasts with other findings (Caraco et al., 1980; Kacelnik and Bateson, 1996, 1997; Abreu and Kacelnik, 1999; Yamada et al., 2013). To be sure, more work is necessary to clarify the effects of related variables such as motivation, “wealth,” and general environmental conditions on risk behavior.

### SAFETY PREFERENCE: EXPERIMENT 7

Despite the risk preference shown in Experiments 1–6 and in published work (McCoy and Platt, 2005; Hayden et al., 2008; O’Neill and Schultz, 2010), the results in Experiment 7 showed that the risk preference could be overcome, and that it was not simply due to factors such as the neglect of likelihood or the insensitivity to trials with no reward. Ultimately, both monkeys demonstrated the capacity to integrate likelihood and value across trials to obtain an expected utility for the risky option, which was lower than the safe option in Experiment 7. The incentive required to induce monkeys to shift from risky to safe options needs further study, especially since our two monkeys appeared to use different strategies. Monkey T used a win-stay, lose-shift strategy, but Monkey P did not. In any case, Experiment 7 shows that rhesus monkeys have the capacity to override any impulsive tendency to select the option with the highest possible outcome under our experimental conditions, as was found in the Yamada et al. (2013) study.

## CONCLUSION

Affective decision-making is a fundamental cognitive process, with multiple phenotypic variations in people (Heino et al., 1996; Kahneman and Tversky, 2000; Weber et al., 2002; Schuck-Paim et al., 2004; Soane and Chmiel, 2005; Paulsen et al., 2011b; Weller and Tikir, 2011), and with significant personal, societal, and clinical ramifications when risk-seeking becomes excessive (Rasmussen and Eisen, 1992; Davis et al., 2004; Fishbein et al., 2005; Kreek et al., 2005; Admon et al., 2012). An evolutionary and comparative analysis should help delineate the nature of its underlying component processes and the conditions under which different phenotypes are manifested.

In the present study, we found that rhesus monkeys prefer risky to safe options in many circumstances, at least when the expected value of the safe and risky options are equivalent. How this risky preference interacts with utility maximization is yet to be determined. Rhesus monkeys are descendants of the last common ancestor (LCA) of Old World monkeys, apes, and humans. Assuming that their capabilities resemble the LCA, at least more closely than modern humans do, this catarrhine species’ abilities may reflect the evolution of cognition in our lineage at a time when both a lower-level impulsive process and a higher-level, more integrative process existed, with perhaps a bias toward the former. Given that a general dual-process architecture is hypothesized to underlie human cognition (Bechara, 2005; Kahneman, 2011; Dolan and Dayan, 2013; Evans and Stanovich, 2013), behavioral and neurophysiological studies with rhesus monkeys should help characterize the dynamics of the underlying cognitive processes, as well as provide insight into the evolutionary history of decision-making in a risky and uncertain world.

## Conflict of Interest Statement

The authors declare that the research was conducted in the absence of any commercial or financial relationships that could be construed as a potential conflict of interest.
